# Disseminated hydatidosis with rare presentation as “cystovesical fistula”

**DOI:** 10.1259/bjrcr.20150222

**Published:** 2016-11-02

**Authors:** Rashmeet Kaur, Simmi Garg, Navkiran Kaur, Paramdeep Singh

**Affiliations:** ^1^Department of Radiodiagnosis, GGS Medical College, Faridkot, India; ^2^Department of Radiodiagnosis, GMC, Patiala, India

## Abstract

Disseminated hydatidosis is a rare disease and may involve any organ of the human body. In this case, an elderly female got infected by *Echinococcus* and presented with disseminated disease. A fistulous communication developed between one of the hydatid cysts present in the retrovesical region and the bladder, because of which the patient presented with hydatiduria.

## Summary

Hydatid disease is a zoonotic infection caused by the tapeworm *Echinococcus granulosus*.^1^ Cystic hydatid disease is a widely distributed endemic parasitosis. Humans become accidental intermediate hosts owing to ingestion of eggs of *E. granulosus,* and rarely of *E. multilocularis* and *E. oligartus*.^2^ In terms of frequency, renal involvement follows hepatic, pulmonary and peritoneal involvement.^3^ Retrovesical hydatid cyst is an unusual entity even in endemic areas. Possible theories on its aetiology include rupture and subsequent seeding in the pouch of Douglas,^4^ haematogenous seeding or direct spread through the rectosigmoid mucosa to the pelvis and perivesical venous plexus.^5^

## Case report

**Figure 1.**
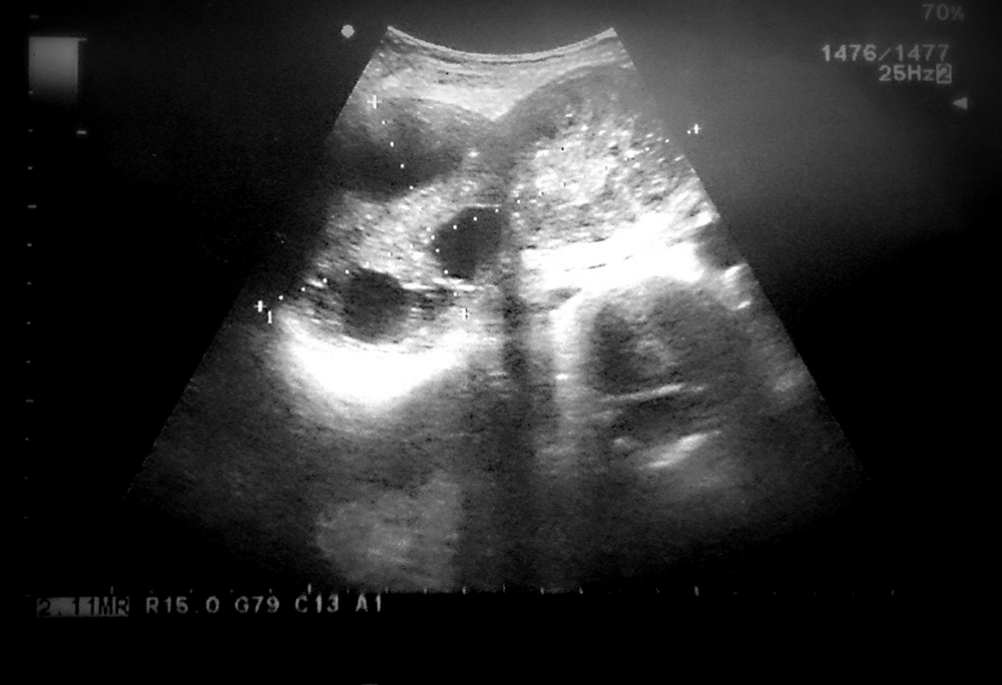
Ultrasonographic imaging showing two adherent heterogeneous lesions in the right hypochondrium, one of which contains multiple daughter cysts arranged at the periphery.

**Figure 2.**
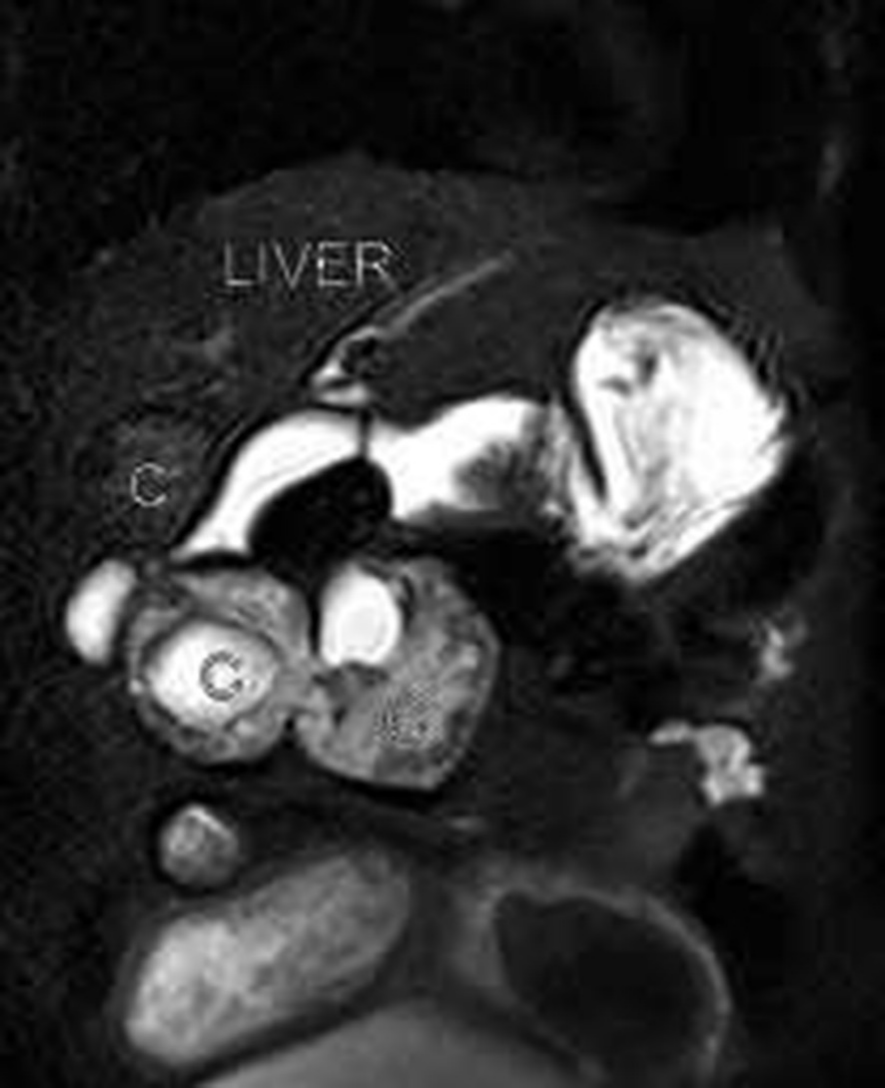
Coronal *T*_2 _MRI showing partially calcified Type III lesion in the liver, which is hypointense, and two intercommunicating, hetrogeneously hyperintense cysts containing daughter cysts in the right hypochondrium.

**Figure 3.**
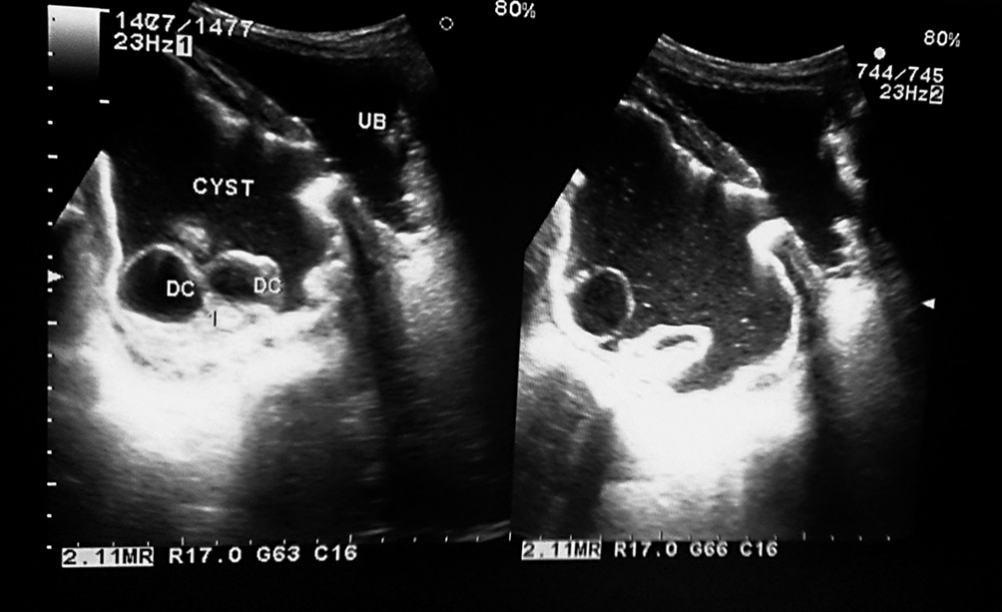
Longitudinal ultrasonographic scan showing fistulous communication between the urinary bladder (UB) and the hydatid cyst containing free daughter cysts (DC).

**Figure 4.**
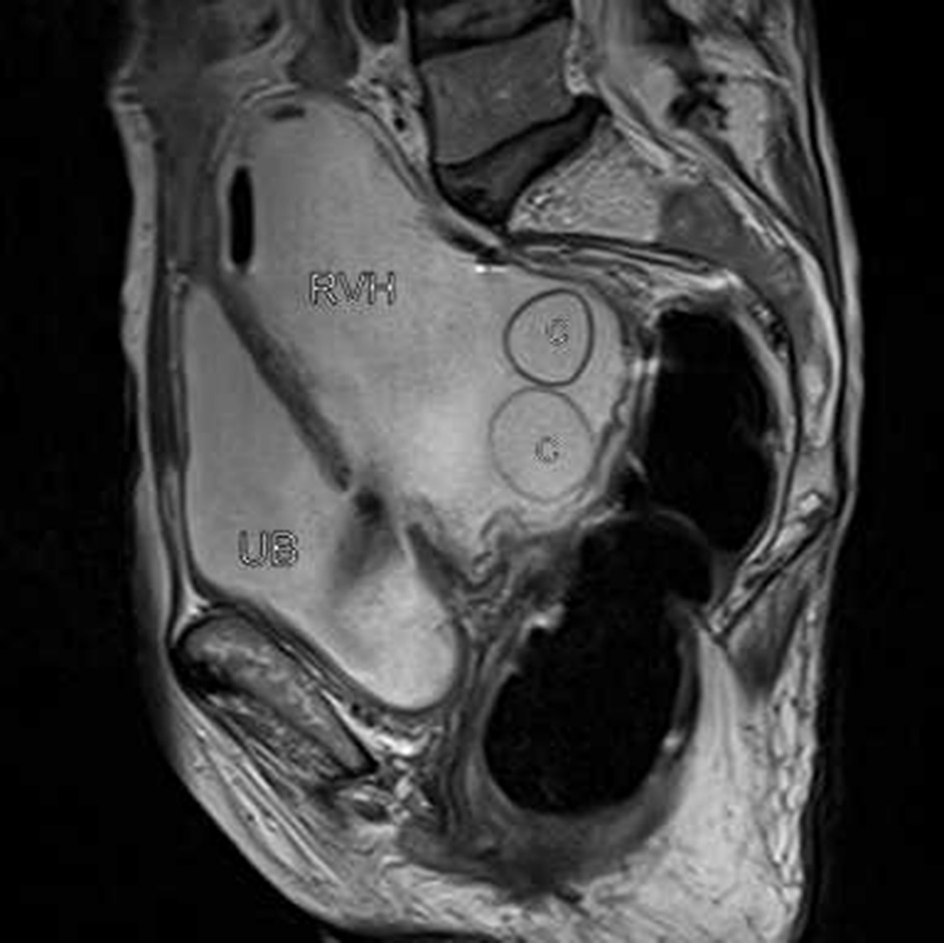
Sagittal *T*_2_ weighted MRI showing a retrovesical cyst (RVH) lying posterior to the urinary bladder (UB) and communicating with it.

A 73-year-old female from a rural region of north India presented with vague abdominal pain, burning micturition and urge incontinence. She gave a history of intermittent passage of small, white, balloon-like, grape-sized structures in the urine for the past 6 months. Her previous medical and family history were unremarkable. Physical examination revealed a visible lump in the epigastric region on the right side and a palpable lump in the left flank. All biochemical and haematological parameters were normal. Chest X-ray was normal. On ultrasonography (USG), two adherent multicystic intraperitoneal lesions were seen occupying the right hypochondrium and the epigastric region (Figure 1), and others were seen in the left lumbar region, right adnexum, right iliac fossa and retrovesical region. On MRI, the lesion in the liver appeared hypointense and those in the hypochondrium appeared as multicystic hyperintense lesions (Figure 2). The large cystic lesion in the retrovesical location contained free-floating daughter cysts and communicated with the posterior wall of the urinary bladder (Figure 3). On MRI, these lesions were characterized as multicystic lesions in respective locations and the retrovesical cyst showed fistulous communication with the urinary bladder (Figure 4). On gross examination of urine, a single balloon-like membranous cyst was seen and histopathological examination showed an outer laminated layer with an inner germinal layer, which was consistent with a hydatid cyst. The serology for hydatid disease was not positive in our case. Based on USG and MRI findings, a diagnosis of disseminated intraperitoneal hydatidosis with hepatic and retrovesical cysts was made. The patient was referred to the urology department for further management.

## Discussion

Intraperitoneal hydatid disease is seen in 13% of all cases of disseminated abdominal hydatidosis.^6^ Dissemination in the peritoneum can occur owing to rupture of already present cysts in other abdominal organs and the cysts can be seen in any peritoneal recess. In the present case, the calcified hydatid cyst was likely the primary site, which led to intraperitoneal involvement. Retrovesical cyst is an unusual entity even in endemic areas and accounts for 0.1–0.5% of hydatid cases. Fewer than 15% of retrovesical hydatid cysts can fistulize, particularly to the bladder,^7,8^ with hydatiduria being a pathognomonic sign of vesicular cystic fistula.^9^ Grape-like material is found in the urine as a result of the cysts rupturing and draining into the urinary tract. Hydatiuria also has been reported in 5–25% of all renal hydatidosis cases. There is no serological and immunological test pathognomonic for hydatid disease.^10^ A negative serological test does not rule out echinococcosis, as our case was also seronegative. Therefore, the test result must always be compared with the radiological findings. The role of plain X-ray in the diagnosis of disseminated abdominal hydatidosis is very limited and non-specific. On X-ray films, only calcified hydatid cysts can be seen as a rim/ring calcified lesion in the respective organs in various quadrants of the abdomen. On the contrary, ultrasound imaging is the main modality used in diagnosing hydatid disease, as it is cheap, non-invasive and easily available. USG helps in the diagnosis of hydatid cysts as the daughter cysts can be demonstrated. On real-time imaging, there is shifting of hydatid sand, appearing as snowstorm in the cyst with postural change of the patient. USG imaging findings range from purely cystic lesions to solid-appearing masses. CT scan has an accuracy of 98% and sensitivity to demonstrate the daughter cysts. Calcification of the cyst wall or internal septa is easily detected on CT scan. The hydatid cysts on plain CT scan are seen as well-defined lesions with or without calcification and with varying attenuation, depending on the contents of the cyst.

On MRI, the hydatid cysts are usually hyperintense on *T*_2_ weighted image but demonstrate a low signal-intensity rim, which has been proposed as a characteristic sign of hydatid disease. Appearance of the hypointense rim is due to the presence of a collagen-rich pericyst, which is generated by host reaction when hydatid cysts are present in the liver.^11^ Also, internal smaller daughter cysts or multicystic appearance of the lesion can be seen on MRI. The fluid-sensitive sequences demonstrate the lesions and help in determining the fistulous communication, as in the present case, owing to the high internal contrast between structures. These cysts can give rise to certain complications, which include rupture leading to anaphylaxis or superinfection, which may lead to increased morbidity and mortality in the patients.

## Conclusions

Hydatid cyst in the retrovesical region is a rare condition and the diagnosis is made by USG and MRI, which help in delineating the exact location of the cyst and its early management. Early diagnosis and optimal choice of surgical management reduce the morbidity and mortality associated with this disease.

## Learning point

1. Hydatiduria can also occur without renal involvement if there is a fistulous communication between the abdominal hydatid cyst and the urinary tract.
2. A negative serology does not rule out echinococcosis, so serology should be correlated with radiological findings.

## Consent

Informed consent has been obtained from the patient.
